# Cricket related hand injury is associated with increased odds of hand pain and osteoarthritis

**DOI:** 10.1038/s41598-020-73586-z

**Published:** 2020-10-08

**Authors:** Karishma Shah, Dominic Furniss, Gary S. Collins, Nick Peirce, Nigel K. Arden, Stephanie R. Filbay

**Affiliations:** 1 Botnar Research Centre, Nuffield Department of Orthopaedics, Rheumatology and Musculoskeletal Sciences (NDORMS), University of Oxford, Old Road, Oxford, OX3 7LD UK; 2 Centre for Statistics in Medicine, University of Oxford, Oxford, UK; 3 England and Wales Cricket Board, and National Centre for Sports and Exercise Medicine and National Cricket Performance Centre, Loughborough University, Loughborough, UK; 4 Centre for Sport, Exercise and Osteoarthritis Research Versus Arthritis, Nuffield Department of Orthopaedics, Rheumatology and Musculoskeletal Sciences (NDORMS), University of Oxford, Oxford, UK; 5 Centre for Health Exercise and Sports Medicine, Department of Physiotherapy, University of Melbourne, Melbourne, VIC Australia

**Keywords:** Rheumatology, Risk factors, Signs and symptoms

## Abstract

Radiographic osteoarthritis (OA) is most prevalent in the hand. The association of hand injury with pain or OA is unclear. The objective was to describe the relationship between hand injury and ipsilateral pain and OA in cricketers. Data from former and current cricketers aged ≥ 30 years was used. Data included history of cricket-related hand/finger injury leading to > 4 weeks of reduced exercise, hand/finger joint pain on most days of the last month, self-reported history of physician-diagnosed hand/finger OA. Logistic regression assessed the relationship between injury with hand pain (in former cricketers) and with OA (in all cricketers), adjusted for age, seasons played, playing standard. Of 1893 participants (844 former cricketers), 16.9% reported hand pain, 4.3% reported OA. A history of hand injury increased the odds of hand pain (OR (95% CI) 2.2, 1.4 to 3.6). A history of hand injury also had increased odds of hand OA (3.1, 2.1 to 4.7). Cricket-related hand injury was related to an increased odds of hand pain and OA. This highlights the importance of hand injury prevention strategies within cricket. The high prevalence of hand pain is concerning, and further research is needed to determine the impacts of hand pain.

## Introduction

Physical activity is known to have a large number of health benefits, and in 2007, the American College of Sports Medicine updated recommendations to encourage increased physical activity participation in the general public^1^. As participation in exercise and sports increases, the prevalence of injury is likely to increase, and sports-related injury is an important item on the research agenda of the Centers for Disease Control and Prevention^2^. Half of the sports-related injuries managed in Orthopaedic departments are to the hand^3^. However, the long-term effects of sports-related hand injury is unclear. In the lower limb, sport-related injury to the knee and hip is an established risk factor for osteoarthritis (OA) development^4–8^. OA has a large personal and economic health burden, and OA treatment costs have been estimated at £579 per person per annum^9^. In the hand, symptomatic OA is prevalent in 7% of American adults, increasing to 27% when diagnosed radiographically^10^, and individuals with hand OA report mental health concerns and poor health-related quality of life^11^.

In the hand, the relationship between injury and OA is not yet well established. The association between finger fracture from any cause and the development of hand OA was investigated by Jones et al.^12^ in 522 Tasmanian adults. They found the odds of radiographic distal interphalangeal joint (DIPJ) OA was increased by two-fold in people with a self-reported history of finger fracture. However, finger fracture was not associated with Heberden’s nodes deformities in the DIPJs. It was also not associated with radiographic first carpometacarpal joint (CMCJ) OA. However, Jones et al.^13^ did not investigate the relationship with symptomatic hand OA. In relation to sports-related hand injury, only one study has investigated the relationship between injury and OA. This study, in retired elite male rugby and cricket players, found no association between severe hand injury and hand OA or pain. However, this study was limited by a small sample and strict inclusion criteria, highlighting the need for further research.

Cricket is a popular world-wide sport with a high prevalence of hand injury^14^. Understanding the relationship between hand injury and OA may encourage the development and implementation of hand injury prevention strategies, which are not common practice amongst sports with a high risk of hand injury. Identifying individuals at high-risk of hand OA could facilitate early investigations and referrals, and targeted treatments to reduce the risk of developing hand OA (for example, weight-loss strategies and education). The aims of this study were to evaluate the relationship between (i) previous hand injury and ipsilateral hand pain on most days of the past month (commonly used as a marker for symptomatic OA^15–17^) in former cricketers, and (ii) previous hand injury and ipsilateral physician-diagnosed hand OA in current and former cricketers.

## Materials and methods

### Procedure

28,152 current and former cricket players, registered on a database managed by the England and Wales Cricket Board, who had agreed to be contacted for cricket-related research, were invited to complete an online epidemiological cross-sectional questionnaire (the Cricket Health and Wellbeing Study (CHWS)). All participants were recruited between March and May 2017. To be eligible to participate in the CHWS, individuals must have been aged 18 years or older and played at least 1 season of cricket. People who were potentially eligible were sent one generic email from the England and Wales Cricket Board, with details of the CHWS, eligibility criteria, and a link to the patient information sheet and consent form. 2548 (9.1%) individuals who received an invitation by email believed they met the eligibility criteria and gave informed consent to participate. Of these, 254 were ineligible and therefore excluded, leaving 2294 participants in the CHWS.

### Ethical approval

The purpose of the CHWS was to evaluate and explain variation in 5 aspects of health and wellbeing in current and former cricketers (i. cricket-related injury; ii. pain and osteoarthritis; iii. general health and disease; iv. physical activity; v. resilience, quality of life and flourishing). This study investigates pain and osteoarthritis in current and former cricketers. Ethical approval was given by the NHS Health Research Authority (NRES), London Stanmore Research Ethics Committee (REC 15/LO/1274). All participants provided informed consent before proceeding to the questionnaire. This study adhered to guidelines and regulations of the Strengthening the Reporting of Observational studies in Epidemiology (STROBE) Statement^18^.

The CHWS questionnaire was designed in collaboration with current and former cricketers, and the England and Wales Cricket Board. The questionnaire was developed on REDCap (Research Electronic Data Capture)^19,20^, hosted at the University of Oxford, with assistance from an experienced database manager. A pilot questionnaire was tested on seven individuals with a range of experience (current and former cricket players, coaching, sports medicine/physiotherapy clinical and research experience). REDCap allowed for branch logic, and participants were able to save their questionnaire progress and resume at a later date. Given the high prevalence of hand injury within cricket, cricketers provide an ideal population to investigate the relationship between hand injury and OA. Data was extracted from the CHWS questionnaire for the current study.

### Eligibility criteria

**Figure 1:**
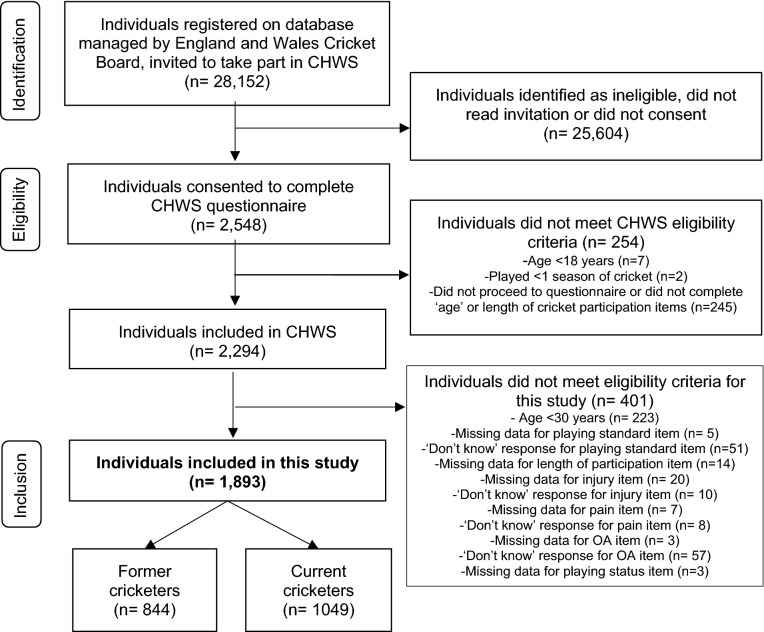
Participant recruitment.

To be eligible for the current study, participants must have been aged 30 years or older at the time of questionnaire completion. Participants with missing data, or ‘don’t know’ responses for joint injury, pain, osteoarthritis, age, playing standard, and length of cricket participation questions were excluded (Fig. 1). Participants who reported using both the right and left hands for bowling and throwing were excluded from a sub-group analyses of dominant and non-dominant hands.

### Outcomes

#### Hand pain

To assess for hand pain, the National Health and Nutrition Examination Survey (NHANES) criteria was used^21^. Participants were asked, ‘Have you had pain in the [left/right] hand or finger(s) on most days of the last month?’, and ‘Yes/No’ response options were offered.

#### Hand OA

To assess for a history of physician-diagnosed hand OA, participants were asked ‘Have you ever been told by a doctor that you have osteoarthritis (wear and tear or joint degeneration) in your hand/finger’, ‘Yes/No’ response options were provided. Those responding ‘Yes’ were then prompted to indicate whether their left, right or both hands were affected.

### Explanatory variables

#### Hand injury

Participants were asked ‘have you ever had any cricket-related [hand/finger] injuries leading to more than 4 weeks of reduced participation in exercise, training or sport?’ (‘Yes/No’ response options). Participants then indicated whether they had injured their left, right of both hand(s).

#### Hand dominance

The dominant hand was defined using the following item, ‘When you bowl or throw do you use your right or left hand?’ (‘Right/Left/Both’ response options). The hand identified was defined as the dominant hand, in the sub-group analyses.

### Confounders

#### Length of cricket participation

Participants were asked ‘Approximately how many seasons have you played cricket for?’ (numerical response).

#### Playing standard

Participants were asked ‘What was the highest standard of cricket that you played for at least one season?’, (response options were: International, County or Premier league, Academy, County-age-group, University, School, Village or Social). Responses were grouped into higher standard (International/County/Premier league/Academy/County-age-group) and lower standard (University/School/Village/Social).

### Statistical analysis

Step-wise logistic regression was used to assess the relationship between hand injury and outcomes (hand pain and hand OA). A hand-level analysis was performed (including both the left and right hands of each participant), whereby the injury status of the left or right hand was assessed in relation to pain/OA in the same (ipsilateral) hand. The relationship between ipsilateral hand injury and hand pain was only assessed in former cricketers, to minimise confounding by pain due to recent cricket-related injury in current players. The relationship between ipsilateral hand injury and hand OA was assessed in all (both current and former) cricketers. Unadjusted and adjusted odds ratios (OR) and 95% confidence intervals (CIs) were calculated for each outcome. All analyses were adjusted for age, number of seasons played, and the highest standard of cricket played (higher/lower standard). All underlying assumptions for logistic regression were checked and satisfied. Due to the small amount of missing data (cricket-related injury: 0.9%, pain: 0.4%, history of OA: 0.1%) and ‘don’t know’ responses (cricket-related injury: 0.9%, pain: 0.3%, history of OA: 2.5%; Fig. 1), a complete case analysis was performed.

#### Sub-group analysis

A sub-group analysis was performed to compare the relationship between ipsilateral hand injury and pain/OA in the dominant and non-dominant hand. Fifteen current players were excluded (10 ambidextrous, 1 ‘don’t known’ response, 4 missing data for hand dominance), and 12 former players were excluded (8 ambidextrous, 4 missing data for hand dominance).

## Results

### Participants

Of the 2294 participants in the CHWS, 401 were ineligible and excluded from the current study (n = 223 aged < 30 years, n = 126 ‘don’t know’ responses, and n = 52 did not complete playing standard/length of participation/injury/pain/OA items). Therefore, 1893 CHWS participants met the eligibility criteria for this study (Fig. 1). Of these, 844 (44.6%) were former cricketers and 1049 (55.4%) were currently playing cricket. The mean age of participants (both current and former cricketers) was 54.7 (standard deviation (SD) 12.1) years, participants had played a mean 30.8 (SD 14.4) seasons of cricket, and 1838 (97.1%) were male. A high standard of cricket was played by 703 (37.1%) participants. The mean age of former cricketers was 60.3 (SD 11.2) years, they had played a mean 30.7 (SD 14.2) cricket seasons, and 316 (37.4%) had played at a high standard (Table 1).

**Table 1:** Participant characteristics.

	Current cricketers n = 1049	Former cricketers n = 844	Hand injury n = 315	No hand injury n = 1578
**Gender**				
Male [N (%)]	1024 (98.1)	814 (97.0)	309 (98.1)	1529 (96.9)
Female [N (%)]	20 (1.9)	23 (2.7)	3 (1.0)	40 (2.5)
Other [N (%)]	0	1 (0.01)	0	1 (0.1)
Do not wish to disclose [N (%)]	0	1 (0.01)	0	1 (0.1)
Missing data [N (%)]	5 (0.5)	5 (0.6)	3 (1.0)	7 (0.4)
**Age (years)**				
Mean (SD)	50.2 (10.9)	60.3 (11.2)	53.9 (12.0)	54.9 (12.2)
Range	30.05–84.25	30.67–93.75	30.25–83.38	30.05–93.75
BMI (kg/m^2^) mean (SD)	27.6 (4.0)	28.7 (5.5)	27.9 (4.7)	28.1 (4.8)
**Smoking status**				
Current [N (%)]	86 (8.2)	55 (6.5)	24 (7.6)	117 (7.4)
Former [N (%)]	146 (13.9)	171 (20.3)	63 (20.0)	254 (16.1)
Never smoked [N (%)]	814 (77.6)	613 (72.6)	225 (71.4)	1202 (76.2)
Missing [N (%)]	3 (0.3)	5 (0.6)	3 (1.0)	5 (0.3)
**Ethnicity**				
Caucasian [N (%)]	941 (89.7)	792 (94.1)	291 (92.4)	1442 (91.4)
Non-Caucasian [N (%)]	96 (9.2)	43 (5.1)	21 (6.7)	118 (7.5)
Do not wish to disclose [N (%)]	9 (0.9)	7 (0.8)	2 (0.6)	14 (0.9)
Missing [N (%)]	3 (0.3)	2 (0.02)	1 (0.3)	4 (0.3)
Length of cricket participation (number of seasons) mean (SD)	30.9 (14.6)	30.7 (14.2)	34.2 (12.4)	30.1 (14.7)
**Highest standard of play**				
Higher (international/county/premiere league/academy/county-age-group) [N (%)]	387 (36.9)	316 (37.4)	138 (43.8)	565 (35.8)
Lower (university/school/village/social) [N (%)]	662 (63.1)	528 (62.6)	177 (56.2)	1013 (64.2)
Prevalence of hand injury [(N (%)]	193 (18.4)	122 (14.5)	N/A	N/A
Dominant hand [(N (%)]	157 (15.2)	90 (10.7)	N/A	N/A
Non-dominant hand [(N (%)]	145 (14.0)	83 (9.8)	N/A	N/A
Prevalence of hand pain [N (%]	84 (8.0)	98 (11.6)	53 (16.8)	129 (8.2)
Physician-diagnosed hand osteoarthritis [N (%)]	31 (3.0)	50 (5.9)	23 (7.3)	58 (3.7)

**The relationship between hand injury and hand pain in former cricketers:** 122 (14.5%) former cricketers reported a history of hand injury leading to more than 4 weeks of reduced participation in exercise, training or sport (dominant hand injury: 90 (10.7%), non-dominant hand injury: 83 (9.8%)). 98 (11.6%) individuals reported hand pain on most days of the last month (11.8% of males and 8.7% of females) (Table 1).

Hand injury was associated with a greater odds of ipsilateral hand pain (OR 2.3, 95% CI 1.5 to 3.6). After adjustment for age, length of participation and playing standard, this relationship remained (2.2, 1.4 to 3.6) (Table 2).

**Table 2:** Logistic regression analysis investigating the relationship between hand injury and ipsilateral hand pain in 844 former cricketers.

	Ipsilateral hand pain	
	OR (95% CI)	*p*
Hand injury	2.3 (1.5–3.6)	*p* < 0.001
Hand injury adjusted for age	2.3 (1.5–3.6)	*p* < 0.001
Hand injury adjusted for age, length of cricket participation	2.4 (1.5–3.9)	*p* < 0.001
Hand injury adjusted for age, length of cricket participation, playing standard	2.2 (1.4–3.6)	*p* = 0.001
**Dominant hand**		
Hand injury	2.1 (1.1–3.9)	*p* = 0.024
Hand injury adjusted for age	2.1 (1.1–4.0)	*p* = 0.023
Hand injury adjusted for age, length of cricket participation	2.2 (1.6–4.3)	*p* = 0.016
Hand injury adjusted for age, length of cricket participation, playing standard	2.0 (1.0–3.9)	*p* = 0.046
**Non-dominant hand**		
Hand injury	2.2 (1.1–4.4)	*p* = 0.028
Hand injury adjusted for age	2.2 (1.1–4.4)	*p* = 0.031
Hand injury adjusted for age, length of cricket participation	2.2 (1.1–4.6)	*p* = 0.028
Hand injury adjusted for age, length of cricket participation, playing standard	2.2 (1.0–4.4)	*p* = 0.039

In the sub-group analysis, hand injury was associated with a greater odds of ipsilateral hand pain in both the dominant (2.1, 1.1 to 3.9) and non-dominant hand (2.2, 1.1 to 4.4). This relationship remained after adjusting for covariates, (dominant hand: 2.0, 1.0 to 3.9, non-dominant hand: 2.1, 1.0 to 4.4) (Table 2).

### The relationship between hand injury and hand OA in current and former cricketers

In all cricketers, 315 (16.6%) individuals reported a history of hand injury (dominant hand injury: 247 (13.0%), non-dominant hand injury: 228 (12.0%). 81 (4.3%) participants had been diagnosed with hand OA (4.4% of males and 2.3% of females) (Table 1). Hand injury was associated with an increased odds of ipsilateral hand OA (2.6, 1.7 to 3.8), and this relationship remained after adjustment for all confounders (3.1, 2.1 to 4.7) (Table 3).

**Table 3:** Logistic regression analysis investigating the relationship between hand injury and ipsilateral hand osteoarthritis (OA) in 1893 current or former cricketers.

	Ipsilateral hand OA	
	OR (95% CI)	*p*
Hand injury	2.6 (1.7–3.8)	*p* < 0.001
Hand injury adjusted for age	2.8 (1.9–4.2)	*p* < 0.001
Hand injury adjusted for age, length of cricket participation	3.2 (2.1–4.8)	*p* < 0.001
Hand injury adjusted for age, length of cricket participation, playing standard	3.1 (2.1–4.7)	*p* < 0.001
**Dominant hand**		
Hand injury	2.2 (1.3–3.9)	*p* = 0.004
Hand injury adjusted for age	2.5 (1.4–4.4)	*p* = 0.001
Hand injury adjusted for age, length of cricket participation	2.8 (1.6–5.0)	*p* = 0.001
Hand injury adjusted for age, length of cricket participation, playing standard	2.7 (1.5–4.9)	*p* = 0.001
**Non-dominant hand**		
Hand injury	2.6 (1.4–4.9)	*p* = 0.003
Hand injury adjusted for age	2.8 (1.5–5.3)	*p* = 0.001
Hand injury adjusted for age, length of cricket participation	3.2 (1.7–6.1)	*p* < 0.001
Hand injury adjusted for age, length of cricket participation, playing standard	3.2 (1.7–6.1)	*p* = 0.001

In the sub-group analysis, hand injury was associated with an increased odds of ipsilateral hand OA in the dominant hand (2.2, 1.3 to 3.9), and non-dominant hand (2.6, 1.4 to 4.9). These relationships remained after adjusting for covariates (dominant hand: 2.7, 1.5 to 4.6; non-dominant hand: 3.2, 1.7 to 6.1 (Table 3).

## Discussion

This is the first study to find a relationship between hand injury and an increased odds of ipsilateral hand pain in former cricketers, and a relationship between hand injury and an increased odds of ipsilateral hand OA in both current and former cricketers. These associations remained after adjustment for age, length of cricket participation, and playing standard. Our results contrast findings from Jones et al., whom found no association between hand injury and pain (on most days of the past month) or a diagnosis of hand OA (using the same criteria as our study)^13^. However, their sample size was only 200 cricket players, with an OA prevalence of 2.4%, and therefore might have been underpowered to detect an effect. Our study included both male and female players, who played at both recreational and elite levels. By using a larger sample size in our study, we might be more likely to identify an association between injury and pain/OA, if one exists.

Due to the cross-sectional nature of our study, we could not imply a causal relationship between hand injury and pain or OA. Similarly, risk factors for the progression of IPJ OA are not yet well understood^22^.  It is possible that other factors could explain the observed relationship. In a recent study involving participants from the CHWS, former elite cricketers were found to have a greater odds of hand pain, compared to former recreational cricketers^23^, and this relationship remained after adjustment for hand injury. We adjusted for playing standard in our analysis, and the positive relationship between hand injury and hand pain/OA remained. Research also suggests that high grip strength is a risk factor for hand OA^24^. Sports such as golf, hockey and tennis demand high grip strength^25^. In these sports, a ‘power’ style grip is used to hold a stick or bat^26^. A similar grip is used by cricketers when batting, a position which all players are required to partake in if an innings is completed. This type of grip exerts remarkably high compressive forces across the hand joints, particularly the proximal interphalangeal joints (PIPJs), the first metacarpophalangeal joint and the first CMCJ^27,28^. These forces might be on the causal pathway for hand OA. However, to our knowledge, the association between grip strength and compressive forces with hand pain and OA have not yet been studied in cricketers. To more accurately assess hand injury as a risk factor for pain and OA, prospective longitudinal studies, adjusted for potential risk factors such as grip strength, are required.

Pain is a common symptom of OA. However, for hand OA, there is not yet established criteria to measure the presence and severity of pain, separate from other symptoms of hand OA. Self-reported questionnaires to measure hand function in patients with hand OA include questions relating to pain^29^. In particular, the Arthritis Impact Measurement 2 Short Form (AIMS2-SF) questionnaire^30^, Score for Assessment and Qualification of Chronic Rheumatoid Affections of the Hands (SACRAH) questionnaire^31^ and the Australian/Canadian (AUSCAN) Osteoarthritis Health Index^32,33^ include questions regarding pain in the hand joints. However, when using these questionnaires, the questions relating only to pain cannot be interpreted separately from other questions. NHANES pain is often used as an OA outcome measure in the lower limb^15–17^. Therefore, in our study, we chose to use NHANES pain in the hand as a surrogate for the prevalence of hand OA. Our results showed similar associations between injury and NHANES pain in the hand, and between injury and hand OA. This suggests that the NHANES criteria for hand pain may be a good marker for symptomatic hand OA. Pain from hand OA has been described as having a diurnal pattern^34^. However, as the NHANES criteria measures pain cross-sectionally, it might be under-reported due to its fluctuating nature. The NHANES criteria also does not differentiate between pain at different joints of the hand, such as an interphalangeal joint (IPJ) and base of thumb. Pain from base of thumb OA can radiate distally to the IPJs, or proximally to the wrist^35^. Therefore, there is a chance the pain measured in our study might not correlate with the anatomical site of injury on the hand. Future research should aim to develop questionnaires to assess pain longitudinally and to differentiate between pain at different anatomical sites, such as by specifying individual joints or by asking about the presence of tenderness when a particular joint is moved.

Hand pain on most days of the past month was reported by 11.6% of former cricketers (11.8% of males and 8.7% of females). Cai et al.^23^ found that the hand was the third most common site where pain was reported on most days of the past month by former cricketers from the CHWS (the most common sites were the knee and back). In a general population study of men and women aged 25–65 years whom were residents of the United Kingdom (UK), the prevalence of hand or wrist pain (defined as pain lasting 1 day or longer in the last 7 days) was 8.7% in men and 11.5% in women^36^. Notably, fewer people are likely to experience hand pain on most days of the past month compared to hand or wrist pain lasting at least 1 day in the last 7 days. Despite this, a greater proportion of former male cricketers reported hand pain compared to males from a general UK population sample. Although our findings suggest hand pain may be less prevalent in female former cricketers compared with the general population, further research is needed due to the small number of females in our study and the heterogeneous criteria used to categorise hand pain.

The high prevalence of hand pain is particularly concerning as pain from hand OA has been described as being as severe as that from rheumatoid arthritis^37^. Hand pain due to OA has also been associated with poor health-related quality of life (HRQoL), and mental health concerns^11^. However, a recent CHWS study in former cricketers found that persistent upper extremity pain was not associated with worse mental component scores (measured with the Short-Form 8 Health Survey), compared to former cricketers without joint pain^38^. Bullock et al.^38^ suggest this might be due to psychological strengths common amongst cricketers, allowing them to cope better with adversity, such as pain. Despite no association with mental component scores, Bullock et al.^38^ found persistent upper extremity pain was associated with worse physical component scores, compared to former cricketers without persistent joint pain, and this exceeded the minimal clinically important difference for this measure. However, no study has specifically looked at the impact of persistent hand pain or hand OA on physical and mental components of HRQoL in former cricketers. It is possible that persistent hand pain in former cricketers has a negative impact on participation in physical activity, work duties or family roles. Qualitative research in former cricketers could provide valuable insights into the physical and mental burden of living with persistent hand pain.

Given the high prevalence of hand injuries reported in this study, there is a need to develop injury prevention strategies for cricket players of all playing standards. In stick-handling sports, the odds of hand/finger injuries and fractures are significantly higher in athletes not using gloves, compared to those using gloves^39^. In cricket, only batsmen and wicketkeepers are permitted to wear gloves. Despite batsmen wearing gloves, Jones et al.^13^ found the highest prevalence of severe hand injury in cricketers was in batsmen. This suggests that the use of personal protective equipment might not be adequate to prevent hand injury in cricketers. Instead, strategies focused on neuromuscular exercise, proprioception and strength training, could be more effective. These strategies have been shown to be successful in lower limb injury prevention^40,41^. However, to our knowledge, injury prevention strategies for the hand have not yet been developed or evaluated. Novel strategies such as these might decrease the incidence of hand injuries, with potential to reduce the burden of symptomatic hand OA.

The participant recruitment strategy was chosen to reach a large, diverse sample of cricketers in the UK. However, a limitation of this recruitment strategy was that we could not determine the response rate or assess for non-response bias. It is, however, possible that cricketers with a particular concern in health and wellbeing were more likely to respond to this questionnaire, introducing a selection bias. Notably, this is a cross-sectional study and all data was collected through self-reported questionnaires, increasing the risk of recall bias. Due to the nature of the questionnaire, we could not reliably assess which hand structures were injured, and it is possible that some injuries did not involve the joints. Similarly, we were unable to account for injuries occurring during other activities. Longitudinal studies are required to more accurately characterise the relationship between hand injuries, confounders and injury/OA. Pain on most days of the past month may be a surrogate for OA in former cricketers^21^. However, in current cricketers where hand injury is prevalent, pain on most days of the past month may reflect an acute hand injury. Since exploring acute pain following hand injury does not align with the objectives of this study, we only investigated the relationship between injury and pain in former cricketers. We assessed hand OA in both current and former cricketers, and despite current cricketers having similar characteristics to former cricketers in our study, it is possible that there were other unmeasured differences between the two groups, which we were unable to account for. Additionally, OA was assessed through a self-reported history of a physician diagnosis. The health-seeking behaviour of cricketers is not well described in the literature, and considering the high rate of persistent hand pain, it is likely that some cricketers had not visited a physician or been assessed for hand OA. It is therefore likely that hand OA is under-reported in this sample.

Additionally, this study did not differentiate between CMCJ OA and (IPJ OA. CMCJ and IPJ OA are thought to be different subsets of hand OA, and therefore might have differing underlying pathologies and aetiologies^42^. Future studies should assess these anatomical sites separately. The number of females in our study was low. However, female gender is known to be associated with incident hand OA^43^, and though previous studies have described injuries sustained by female cricketers^44,45^, further work is needed to better understand the association between hand pain and OA with injury in female cricketers.

## Data Availability

The datasets generated during this study are available from the senior author on reasonable request.
